# Bladder Base Displacement during Abdominal Muscles Contraction and Functional Activities in Primiparous Women Assessed by Transabdominal Ultrasound: A Descriptive Study

**DOI:** 10.3390/jcm11010025

**Published:** 2021-12-22

**Authors:** Beatriz Arranz-Martín, Patricia García-Gallego, Helena Romay-Barrero, Beatriz Navarro-Brazález, Carlos Martínez-Torres, María Torres-Lacomba

**Affiliations:** 1 Physiotherapy in Women’s Health (FPSM) Research Group, Physiotherapy Department, Faculty of Medicine and Health Sciences, University of Alcalá, 28805 Madrid, Spain; beatriz.arranz@edu.uah.es (B.A.-M.); patri_gg97@hotmail.com (P.G.-G.); carlosmt888@gmail.com (C.M.-T.); maria.torres@uah.es (M.T.-L.); 2 Department of Nursery, Physiotherapy and Occupational Therapy, Faculty of Physiotherapy and Nursery, University of Castilla-La Mancha, 45071 Toledo, Spain; Helena.Romay@uclm.es

**Keywords:** abdominal muscles, abdominopelvic cavity, intra-abdominal pressure, pelvic floor muscles, postpartum period, ultrasonography, urinary incontinence

## Abstract

This study described the response of the bladder base (BB) by transabdominal ultrasound in primiparous women during movements that activate the abdominopelvic cavity musculature and cause variations in intra-abdominal pressure (IAP). A descriptive cross-sectional study was conducted in 64 primiparous women at eight weeks after uncomplicated delivery. BB displacement was measured using a 5-MHz convex transducer in a suprapubic position. Participants were asked to perform the isolated contraction of pelvic floor musculature (PFM) and transverse abdominis (TrA), cough at high lung volume and trunk flexion with and without maximal voluntary contraction of PFM. PFM contraction elevated the BB in all but one participant, whereas TrA contraction caused the BB to ascend in 56% of the women and descend in the rest; their combined contraction rose the BB in 65% of the women although the effect was greater with only PFM contraction (*p* < 0.01). The BB descended in all participants during coughing and trunk flexion although the descent was inferior with the joint maximal voluntary contraction of PFM (*p* < 0.01). In conclusion, TrA contraction must be assessed individually in puerperal women since its effect on the BB varies among subjects. During movements increasing IAP, such as coughing or curl-ups, the anticipatory contraction of PFM reduces bladder descent although not sufficiently to counteract bladder displacement.

## 1. Introduction

The pelvic floor is comprised of a complex network of muscles, ligaments, and fasciae that act as a functional unit and form the basis of the abdominopelvic cavity [1]. The physiological, hormonal, and mechanical changes from pregnancy and childbirth appear to increase the likelihood of musculoskeletal, conjunctival, and/or nerve injuries of the pelvic floor [2,3].

During vaginal delivery, about 80% of women present some kind of morpho-functional alteration of the pelvic floor musculature (PFM), especially the levator ani [4]. This may be due to the mechanical hyper-solicitation to which the PFM is subjected [2,5,6], with an increase of up to 20–30% in the size of the urogenital hiatus and increased urethral mobility, which predisposes to the early or late onset of pelvic floor dysfunctions (PFD) [5].

PFD is a group of disorders resulting from an alteration in the integrity and/or function of the PFM, including urinary incontinence (UI), anal incontinence, pelvic organ prolapses, and symptoms of sexual dysfunction [2,3,4,7]. They constitute a well-known health problem affecting 25–30% of the adult female population and reaching up to 50% after labor, having significant repercussions on quality of life and affecting the health, social, and economic spheres [2,3,8].

The etiology of PFD is complex and multifactorial, with numerous factors related to its appearance, such as gender, aging, obesity, and menopause [9]. However, the factors most strongly affecting the progression of PFD appear to be pregnancy and vaginal delivery [4,5,10] as well as physiological and pathological conditions associated with increased intra-abdominal pressure (IAP), such as constipation, coughing, and high-impact activities [6,11].

IAP is defined as the physiological load that is transmitted through the entire abdominopelvic cavity and that fluctuates according to the activity exerted, causing modifications in the mechanical load of the pelvic floor [6]. The correct functioning of this cavity and the proper management of IAP depend on the coordinated action and synergies between the muscles that constitute it, such as the PFM and abdominal muscles [12], as well as the automatic adaptive and anticipatory response mechanisms of the PFM to the increases in IAP generated in daily activities [13].

During the puerperium, a process of tissue and biomechanical remodeling of the PFM takes place, resulting in decreased tone, strength, neuromuscular activity, and resistance to stretching [2,14]. Hence, puerperium is considered to be the time of greatest vulnerability for the pelvic floor and the onset of PFD [15], with stress UI being the most frequent. This may be due to both deficient activity of the PFM and the consequent alteration of abdominopelvic synergy when counteracting increases in IAP [16]. In this context, the knack maneuver, consisting of the maximum voluntary contraction (MVC) of the PFM performed immediately before and during an increase in IAP, must be highlighted as an effective measure to prevent and manage stress UI, whose mechanism of action appears to be urethral closure and stabilization of the urethra and bladder neck [17].

Electromyographic research has demonstrated the presence of patterns of coactivation and synergy between the muscles of the abdominopelvic cavity [18,19] in healthy and nulliparous populations [8]. However, these studies do not allow the evaluation of muscle synergy in relation to the pressures generated in the abdominopelvic cavity and their impact on visceral structures or structures supporting the pelvic floor, such as the position of the bladder base. Functional transabdominal ultrasound is a non-invasive, valid, and reliable method for real-time objective evaluation of the dynamic relationship of the PFM, the contraction of the abdominal musculature, and the mechanical effect of IAP alterations on the supporting structures of the pelvic floor, especially when faced with functional activities that involve an increase in IAP [7,8].

The purpose of this study was to describe by means of functional transabdominal ultrasound the response of the bladder base in postpartum women to movements and functional maneuvers that can generate the activation of the abdominopelvic cavity musculature and variations in IAP. We hypothesized that the voluntary contraction of the PFM would result in an elevation of the bladder base, while abdominal contraction maneuvers would generate a downward thrust. Therefore, the present study aimed to know the bladder displacement by transverse and sagittal transabdominal ultrasound during the following exercises: (i) PFM contraction, (ii) transverse abdominis (TrA) muscle contraction, (iii) TrA muscle contraction together with PFM contraction, (iv) coughing, (v) coughing combined with PFM contraction, (vi) trunk flexion or curl-up, and (vii) curl-up plus PFM contraction.

## 2. Materials and Methods

### 2.1. Study Design

A cross-sectional descriptive study was conducted between November 2018 and May 2019.

The Ethics Committee for Clinical Research of the Hospital Príncipe de Asturias approved the trial under number (OE 21/2013), which followed the guidelines of the Declaration of Helsinki.

All subjects provided informed consent. Anonymity and confidentiality were guaranteed through the encoding of files, complying with current regulations and the Declaration of Helsinki.

### 2.2. Participants

Postpartum women referred for follow-up in the postpartum stage from the Hospital Universitario Príncipe de Asturias of Alcalá de Henares (Madrid) to the Physiotherapy in Women’s Health Research Group (FPSM group) of the Universidad de Alcalá.

All primiparous women in the eighth week of the puerperium who had had an uncomplicated vaginal delivery of a single child and who freely accepted and signed the informed consent to participate in the study were included. The exclusion criteria were: being a minor (<18 years of age), being pregnant, presence of urinary or vaginal infection, and having cognitive or language limitations that prevented understanding of the study procedures.

### 2.3. Procedure

A physical therapist (MTL) specialized in women’s health with more than 20 years of experience collected the following demographic and clinical data from the participants: age, height, weight, pregnancy and childbirth history, current medication, physical activity practice, and the presence of PFD symptoms as assessed through the Pelvic Floor Distress Inventory–Short Form 20 (PFDI-20) and Pelvic Floor Impact Questionnaire–Short Form 7 (PFIQ-7) [20]. Subsequently, the strength and capacity for voluntary contraction of the PFM were evaluated by two fingers placed in the vagina and quantified through the modified Oxford scale (MOS). The contraction of the adjacent musculature during PFM contraction, presence of inspiratory apnea, appearance of PFM reflex during coughing, and ability to maintain a voluntary contraction of the PFM during coughing were also recorded.

Subsequently, another physiotherapist (BAM) specialized in women’s health with more than 5 years of experience performed the transabdominal ultrasound assessment. The initial position of the participants was supine decubitus with the head on a pillow and lower limbs semi-flexed and comfortably resting on a roller. First, the abdominal musculature was assessed during the MVC maneuvers of the PFM, instructing the participant to “clench your pelvic floor muscles around the urethra, anus, and vagina, as if holding the urine and gas, as hard as you can, without contracting the muscles of the abdomen or legs”. The isolated contraction of the TrA was assessed by instructing the participant to “push the navel inward, as if you wanted to stick it to the back, without moving the pelvis” [18]. These measurements allowed detecting the synergy, or lack thereof, of the abdominal musculature with the voluntary contraction of the PFM, and to check the correct isolated contraction of the TrA. An ultrasound scanner Mindray M7 and a linear transducer L14-6Ns (Mindray, Shenzhen, China) were employed with a frequency of 12 MHz. To evaluate the external oblique, internal oblique, and TrA muscles, the probe was placed perpendicular to the right anterolateral abdominal wall, oriented transversely between the iliac crest and the costal angle at the prolongation of the right axillary line [21]. To assess the rectus abdominis muscle, the probe was placed perpendicular to the abdomen, at a point 2 cm above the navel and 2 cm lateral to the midline, at the center of the muscle belly [21,22]. Subsequently, bladder displacement was assessed in the transverse plane and then in the sagittal plane. To ensure adequate visibility of the bladder, participants were asked to urinate for the last time one hour before the assessment and to drink 500 mL of water half an hour before starting the test. A convex C5-2s probe (Mindray, Shenzhen, China) with a frequency of 5 MHz was employed in motion mode M. The scan in the transverse plane was performed with the probe immediately above the pubic bone in the median plane. To obtain correct angulation of the probe, a contraction of the PFM was requested in order to determine an angle of 15–30° to obtain a clear image of the bladder base displacement [21]. Ultrasound assessment in a sagittal view was then performed with the probe placed on the linea alba, at the suprapubic level, and with a caudal inclination of the probe to allow the correct visualization of the vagina and the bladder base. Two repetitions of the following exercises were performed in transverse and sagittal views: (1) MVC of the PFM; (2) isolated contraction of the TrA; (3) MVC of the PFM together with TrA muscle activation; (4) cough at high lung volume, following a prompt to “cough hard”; (5) sustained MVC of the PFM followed by cough at high lung volume; (6) curl-up, following request to “raise your head and shoulders until the lower edge of the scapula is separated from the stretcher”; and (7) sustained MVC of the PFM followed by a curl-up.

### 2.4. Data Collection

Ultrasound measurements of the abdomen were performed in B mode. The thickness of the abdominal muscle bellies was calculated as the distance between the upper and lower hyperechoic fasciae limiting the musculature. The thickness of the muscle bellies at the end of normal expiration was considered as the reference thickness [23]. Mindray’s software calipers were used to evaluate the change in the TrA thickness during MVC of the PFM.

**Figure 1:**
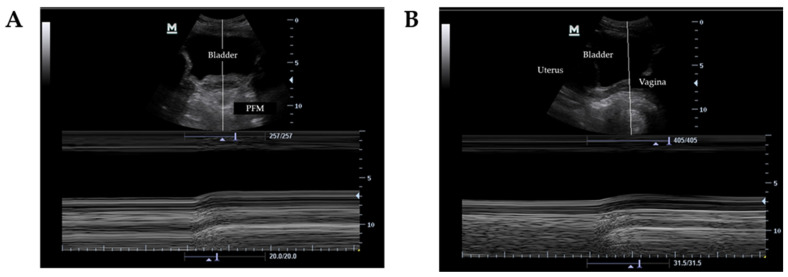
Ultrasound of the bladder base displacement in motion mode M. (**A**) MVC of PFM in transversal plane. (**B**) MVC of PFM in sagittal plane.

**Figure 2:**
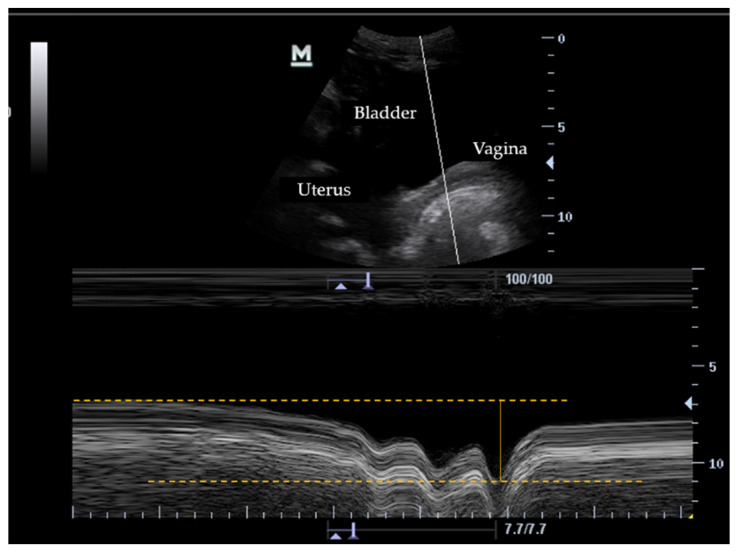
Ultrasound measurements of the bladder base displacement during a cough in sagittal plane. In the top of the figure pelvic structures are shown. Below is represented the timeline of M mode. The dotted horizontal lines represent the bladder base in rest position and the position during a cough. The vertical continued line shows the bladder displacement.

Ultrasound measurements of the PFM were performed in motion mode M using Mindray’s calipers. In the transverse plane, the lower line of the bladder was used as a reference (marked by the end of the anechoic margin and the beginning of the hyperechoic line representing the deep plane of the pelvic floor) (Figure 1A). In the sagittal plane, the posterior margin of the bladder (represented by the hyperechoic line of the vagina after the anechoic image of the bladder) was taken as a reference (Figure 1B). Two repetitions of each programmed exercise were performed, and the average difference between the maximum displacement of the bladder base and the initial position of the bladder base with the PFM at rest was obtained [8] (Figure 2).

### 2.5. Sample Size Calculation

The study was designed to detect a difference equal to or greater than 0.2 units between the displacements during coughing and coughing with pelvic floor contraction assuming a standard deviation (SD) of 0.75. This a-priori sample size estimation was calculated based on the findings of a previous pilot study conducted ad hoc to test the methods and estimate the sample size. Finally, for an alpha error of 0.1 and a beta error of 0.2 in unilateral contrast, a total of 64 participants were recruited. Sample size was calculated using the statistical Granmo 7.12 (Institut Municipal d’Investigació Mèdica, Barcelona, Spain, 2012).

### 2.6. Statistical Analyses

Statistical analyses were performed using IBM SPSS Statistics for Windows, v.24 (IBM Corp., Armonk, NY, USA). Quantitative variables were described by their arithmetic mean (ẋ) and SD or by their median (M_e_) and interquartile range (IQR), depending on whether or not the variable fit a normal distribution, as determined by the Shapiro–Wilk (S-W) statistical test. Categorical variables were expressed by their absolute and relative percentage frequencies. Student’s *t*-test for paired samples was used to determine the association between dichotomous independent and quantitative dependent variables, obtaining the difference of means and considering a 95% confidence interval in the case of normal variables, whereas the Wilcoxon test for paired samples was employed for non-normal variables, calculating the difference of medians. Statistical significance was set at *p* < 0.05.

## 3. Results

Sixty-four women participated in the study (Table 1), of which 82% presented a score ≥3 on the MOS, and 74% reported at least one symptom of PFD, with UI being the most frequent (Table 2).

Regarding the behavior of the abdominal musculature during MVC of the PFM, increased TrA muscle thickness was found in 73% of the women with a mean of 0.33 cm (SD 0.08) (Table 3).

MVC of the PFM elevated the bladder base in 63 participants in the transversal plane and 62 women in the sagittal plane, with median values of 0.41 (IQR 0.64-0.29) cm and 0.39 (IQR 0.55–0.21) cm, respectively. Isolated contraction of the TrA elevated the bladder base in 56% and 25% of the participants in the transverse and sagittal planes, respectively, but the bladder base descended in 37.50% and 44.44% of women in the transverse and sagittal planes, respectively. This descent was counteracted when the MVC of the PFM was added to the contraction of the TrA although the bladder base continued to be pushed down in 30.51% of the sample. Bladder base elevation during MVC of the PFM was greater than those recorded during the isolated contraction of the TrA muscle and with the combined contraction of the PFM and TrA (Table 4).

A caudal displacement of the bladder base occurred during the maneuvers of coughing at high lung volume and curl-up when performed both with or without MVC of the PFM (Table 4). With the addition of MVC of the PFM, the displacement was inferior (*p* < 0.01) before and during these practices for the majority of women. However, greater bladder descent was observed in 8% and 17% of the participants when combining the PFM contraction with cough or curl-ups, respectively.

## 4. Discussion

To the authors’ knowledge, this is the first study to describe by functional transabdominal ultrasound the response of the bladder base in primiparous women, some with symptoms of PFD, to different muscle contraction exercises (MVC of the PFM, the contraction of the TrA muscle, or curl-ups) as well as the response to daily actions that can increase IAP (e.g., coughing). Bladder base elevation was observed in all but two women during MVC of the PFM and in approximately half of the sample during both the isolated TrA muscle contraction and the combined contraction of the PFM and TrA. However, bladder descent was detected in all women during coughing and curl-ups.

Transabdominal ultrasound has shown to be a valid and reliable tool for assessing PFM function, with high intrarater and interrater reliability [21]. In addition, this ultrasound modality is frequently used for measuring the cross-sectional area of the abdominal musculature, where it shows good interrater reproducibility in measuring the thickness of both the lateral [24] and anterolateral [23] abdominal musculature in healthy subjects.

Expiring at high lung volumes, as occurs during coughing or sneezing, and high-impact activities or those involving the concentric activation of the abdominal muscles, such as curl-ups, can lead to increased IAP [11]. This increased IAP causes the caudal and anterior displacement of the bladder neck and therefore the potential distension of the connective tissue of the pelvic floor [6]. Under normal conditions, there is an adaptation mechanism of the PFM for adequate continence control that helps to counteract this displacement, favoring urethral closure and the correct position of the bladder neck [25]. Pregnancy and childbirth subject the PFM to changes that can affect these adaptation mechanisms and cause a possible delay in the activation of the PFM in the face of an increase in IAP, which makes pregnancy and vaginal delivery etiological factors of PFD that strongly correlate with stress UI [25,26]. The present study found that the bladder base was displaced caudally in 100% of the participants when coughing at high lung volume, while the appearance of a contraction reflex of the PFM was found in only 36% of the participants, as assessed by intravaginal palpation. The absence of anticipatory contraction of the PFM during coughing together with the observed bladder descent may be related to the high prevalence of PFD symptoms (74%) in this population of primiparous women, with UI (46.9%) being the most frequent. The prevalence of UI found in this study coincided with that described by Ahlund et al. [27] in primiparous women with uncomplicated delivery one year after delivery, which reported that stress UI is the most prevalent PFD in the puerperium, with no relationship found between its occurrence and obstetric factors, such as duration of the delivery, infant weight, or infant’s head circumference. Other studies have associated the appearance of stress UI in the puerperium with the presence of symptoms before or during pregnancy and with obesity [28]. However, the women included in the present study were normal weight, and only 3.12% reported previous stress UI.

The bladder descent during curl-ups was similar to that observed in other studies. Martinez-Bustelo et al. [29] evaluated the effect of curl-up exercises on the bladder base in continent women. They found a descent of −0.66 (SD 0.51) cm in nulliparous women, a figure similar to that recorded in our sample when associated with PFM contraction (−0.62 cm; SD 0.84), and −1.31 (SD 0.57) cm in postpartum women, which was slightly lower than the −1.51 (SD 0.66) cm observed in our study. In agreement with their findings, the present study also concluded that repeated curl-up exercises during postpartum could be a risk factor for the development of PFD, especially in postpartum women with former symptoms of PFD.

Based on our outcomes, the anticipatory and maintained contraction of the PFM, a maneuver termed knack, during coughing at high lung volume and curl-up exercises produces a significant decrease in bladder descent (*p* < 0.01). This is consistent with previous studies that have shown how the knack reduces bladder neck mobility and aids in the control of urinary leaks [17]. However, this contraction of musculature combined with coughing and curl-ups increased the push of the bladder base in two participants, highlighting the need for prior training of the knack maneuver to ensure its correct performance [30]. These inter-subject differences have been particularly prominent in the bladder base response and TRA muscle contraction. Previous studies have evaluated the muscle synergy between the TRA and PFM [13,18,31,32,33]. MVC of the PFM in healthy women has been associated with the activation of the TRA and internal oblique muscles and inversely correlated with the onset of muscle activity in the PFM during the isometric contraction of the abdominal musculature [13,33]. However, this relationship that is present in healthy women could be altered after pregnancy and vaginal delivery [34] as well as in women with PFD. Additionally, the studies that show beneficial synergy between deep abdominal musculature and the PFM employed electromyography for assessment and did not consider the potential effect of an increase in IAP on the position of the bladder base. In this regard, the results of the present study agree with those obtained by Bø et al. [35] in a study performed in 20 continent women. They found that voluntary contraction of the PFM produced a bladder base elevation in 19 of 20 women, while the isolated contraction of the TrA muscle caused a bladder base descent in 30% of the subjects, from which they concluded that the bladder elevation was 61.6% greater with the PFM contraction than with the isolated contraction of the TRA muscle. The present study found statistically significant differences (*p* < 0.01) in bladder elevation in favor of MVC of the PFM versus TrA muscle contraction. Other two studies also analyzed the response of the bladder base [29] by transabdominal ultrasound and the bladder neck [36] by transperineal ultrasound during submaximal PFM contractions. Both studies reported that the moderate contraction of the TrA muscle produced an elevation of the bladder base and bladder neck similar to that obtained by moderate contraction of the PFM. Differences with our study may be due to the intensity of muscle contractions as well as differences in the evaluated population since their studies did not include women with PFD. Although moderate contractions of the PFM may be more common based on its histologic composition [37], therapy involving maximal contractions of the PFM has shown its effectiveness in preventing and correcting symptoms of urinary and anal incontinence in postpartum women [38].

In terms of limitations of the study, causal relationships between the variables cannot be established due to its descriptive design. The study population was limited to primiparous women between six and eight weeks postpartum, and we cannot extrapolate the found results to nulliparous women or to women with pelvic pain, whose PFM behavior may be different during abdominal muscle contractions or during IAP increases. Furthermore, all women came from a single hospital, which limits external validity. The main limitation related to the measuring instrument is that transabdominal ultrasound of the PFM uses a potentially mobile starting point [21], so the skills of the examiner are of key importance. In addition, as bladder filling is necessary, contraction and relaxation of the PFM can be complicated, especially in women with UI [39], and bladder visibility from the abdominal approach can be difficult in overweight women [40].

## 5. Conclusions

MVC of the PFM produced an elevation of the bladder base in primiparous women. This elevation may also appear during maximum contraction of the TrA muscle as well as during the combined contraction of PFM and TrA. However, both maneuvers produced a descent of the bladder base in some women, so TrA muscle contraction should be assessed individually in puerperal women before their inclusion in a therapy program.

Conversely, coughing at high lung volume and curl-up exercises produced a bladder base descent in puerperal women that can be minimized but not eliminated by the early and maintained contraction of the PFM. Therefore, curl-up exercises should be avoided in the postpartum period, especially in women with PFD symptoms, while women should be trained in knack procedures to ensure their correct performance. More studies are needed to evaluate the PFM response during abdominal muscles contraction or IAP maneuvers in nulliparous women and in women with pelvic pain since other muscle synergies can be found. Furthermore, it would be desirable more studies evaluating the functional relationships between the PFM, the abdominal muscles, and IAP due to the inter-observer reliability implicit in the use of transabdominal ultrasound.

## Figures and Tables

**Table 1:** Demographic characteristics of participants.

Characteristics	*n* = 64
Age (years), ẋ (SD)	35 (6)
BMI (Kg/m^2^), ẋ (SD)	23.93 (4.38)
Weeks of gestation, ẋ (SD)	40 (3)
Labour duration (hours), ẋ (SD)	10 (5)
Episiotomy, *n* (%)	51 (80)
Childbirth preparation, *n* (%)	62 (97)
Baby weight (Kg), ẋ (SD)	3.16 (0.44)
Medication, *n* (%)	
None	8 (13)
Iron and/or iodine	52 (81)
Laxatives	1 (2)
Antihypertensives	3 (5)
Smoker, *n* (%)	5 (8)
Respiratory/allergy pathology, *n* (%)	15 (23)
Chronic cough, *n* (%)	0 (0)
Constipation, *n* (%)	31 (48)
Physical activity, *n* (%)	
None	8 (12)
Low impact	38 (59)
High impact	13 (20)
High and low impact	5 (8)

ẋ, mean; SD, standard deviation; BMI, body mass index; *n*, number.

**Table 2:** Clinical characteristics of participants.

Characteristics	*n* = 64
Symptoms of PFD after delivery, *n* (%)	47 (73)
UI	30 (46.90)
Stress urinary incontinence	18 (28.10)
Urgency urinary incontinence	4 (6.30)
Mixed urinary incontinence	8 (12.50)
AI	13 (20.30)
Fecal incontinence	1 (1.56)
Gas incontinence	12 (18.70)
Vaginal lump	1 (1.56)
PFD before/during pregnancy	2 (3.12)
Manual assessment of PFM with MOS, *n* (%)	
MOS = 0	1 (1.56)
MOS = 4	18 (28)
MOS = 5	1 (1.56)
Contraction reflex of PFM during coughing, *n* (%)	23 (36)
PFM contraction sustained during coughing, *n* (%)	38 (60)
Accessory muscles, *n* (%)	
Abdominal	7 (10.94)
Gluteus	10 (15.63)
Adductors	2 (3.13)
Abdominals and gluteus	5 (7.81)
Abdominals and adductors	1 (1.56)
Abdominals, gluteus, and adductors	5 (7.81)
None	34 (53.12)
Apnea inspiratoria, *n* (%)	47 (73.44%)

*n*, number; PFD, pelvic floor dysfunctions; UI, urinary incontinence; AI, anal incontinence; PFM, pelvic floor muscles; MOS, modified Oxford scale.

**Table 3:** Characteristics of the participating women: transversal section of abdominal musculature during the physiotherapeutic assessment via functional ultrasound.

Muscle	*n* = 64	*p*-Value
RA at rest (cm), M_e_ (IQR)	0.73 (0.82–0.66)	
EO at rest (cm), ẋ (SD)	0.44 (0.11)	
IO at rest (cm), M_e_ (IQR)	0.55 (0.62–0.45)	
TrA at rest (cm), M_e_ (IQR)	0.27 (0.34–0.24)	
TrA during MVC of PFM (cm), ẋ (SD)	0.33 (0.08)	<0.01 ^W^ *

M_e_, median; IQR, interquartile range; ẋ, mean; SD, standard deviation; RA, rectus abdominis muscle; EO, external oblique muscle; IO, internal oblique muscle; TrA, transverse abdominis muscle; MVC, maximal voluntary contraction; PFM, pelvic floor muscles. ^W^ Wilcoxon test. * statistically significant.

**Table 4:** Displacement of bladder base during physiotherapy assessment with functional ultrasound.

	Displacement of Bladder Base						Women with Caudal Displacement of Bladder Base, *n* (%)	
	Transversal Plane			Sagittal Plane				
Measurements	Displacement of bladder base (cm) ẋ (SD)/M_e_ (IQR)	CI 95%Z	*p*-Value	Displacement of bladder base (cm) ẋ (SD)/M_e_ (IQR)	CI 95%	*p*-value	Transversal plane	Sagittal plane
MVC of PFM	0.41 (0.64–0.29)		<0.01 ^W^ *	0.39 (0.55–0.21)		<0.01 ^W^ *	1 (1.56)	2 (3.13)
TrA contraction	0.09 (0.24–(−0.25))			0.01 (0.24–(−0.29))			24 (37.50)	28 (44.44)
MVC of PFM + TrA	0.25 (0.42–(−0.22))		<0.01 ^W^ *	0.1 (0.42–(−0.06))		<0.01 ^W^ *	18 (30.51)	18 (30.51)
Cough (cm)	−3.11 (1.15)	−0.97–0.53	<0.01 ^S^ *	−3.23 (1.10)	−0.91–(−0.55)	<0.01 ^S^ *	64 (100)	64 (100)
Cough + MVC of PFM	−2.36 (1.02)			−2.05 (0.97)			64 (100)	64 (100)
TF (cm)	−1.51 (0.66)	−0.89–(−0.53)	<0.01 ^S^ *	−1.30 (0.72)	−0.77–(−0.47)	<0.01 ^S^ *	64 (100)	63 (98.44)
TF + MVC of PFM	−0.62 (0.84)			−0.68 (0.64)			57 (89.06)	57 (89.06)

MVC, maximal voluntary contraction; PFM, pelvic floor musculature; M_e_, median; IQR, interquartile range; TrA, transverse abdominis muscle; ẋ, mean; SD, standard deviation; TF, trunk flexion; CI, confidence interval; ^S^, Student’s *t*-test and ^W^ Wilcoxon test. * statistically significant.

## Data Availability

Data are kept securely by the research team and may be available upon reasonable request and with relevant approvals granted.
